# Soil microbial carbon utilization, enzyme activities and nutrient availability responses to *Bidens pilosa* and a non-invasive congener under different irradiances

**DOI:** 10.1038/s41598-017-11707-x

**Published:** 2017-09-12

**Authors:** Hui Wei, Wenbin Yan, Guoming Quan, Jiaen Zhang, Kaiming Liang

**Affiliations:** 1 Department of Ecology, College of Natural Resources and Environment, South China Agricultural University, Guangzhou, 510642 China; 2 Key Laboratory of Agro-Environment in the Tropics, Ministry of Agriculture, Guangzhou, 510642 China; 3 Guangdong Engineering Research Center for Modern Eco-agriculture and Circular Agriculture, Guangzhou, 510642 China; 4 Department of Urban Construction Engineering, Guangzhou City Polytechnic, Guangzhou, 510405 China; 5 The Rice Research Institute of Guangdong Academy of Agricultural Sciences, Guangzhou, 510640 China

## Abstract

Two Bidens species (*Bidens pilosa* and *B. bipinnata*) that originate from America have been introduced widely in pan-tropics, with the former regarded as a noxious invasive weed whereas the latter naturalized as a plant resource. Whether the two species exhibit different effects on the belowground system remains rarely studied. This study was conducted to investigate soil microbial carbon (C) utilization, enzyme activities and available nitrogen, phosphorus and potassium contents under the two species in a subtropical garden soil of southern China under different levels of light intensity. Results showed that the microbial C utilization and enzyme activities were not significantly different under the two species, implying that the strong invasiveness of *B. pilosa* could not be due to the plant-soil microbe interactions, at least plant-induced alterations of microbial community function to utilize C substrates. Alternatively, available soil nitrogen and potassium contents were significantly higher under *B. pilosa* than under *B. bipinnata* in full sun, indicating that the strong invasiveness of *B. pilosa* could result from rapid nutrient mobilizations by *B. pilosa*. However, the differences turned non-significant as light intensity decreased, suggesting that light availability could substantially alter the plant effects on soil nutrient mobilizations.

## Introduction

Biological invasion has received growing concerns, because a deal of previous studies reported its negative effects on ecosystem properties^1, 2^. Alien plants can greatly change ecosystem properties and ecological processes such as soil carbon (C)/nitrogen (N) cycling, soil microbial community, and soil organic matter and litter decomposition in invaded habitats, consequently influencing stabilization of ecosystem functions^3, 4^. Hundreds of million dollars are spent to prevent from and control biological invasion each year^5^, but it remains a global environmental issue because of its harms to native species and environment in invaded ecosystems. Therefore, considerable attentions have been focused to clarify the underlying invasion mechanisms, to observe invasion consequences and to explore effective control measures, aiming to minimize and diminish invasion effects. Nevertheless, many aspects of invasion are not completely understood yet and it remains difficult to drawn a general conclusion according to the existing knowledge^1, 4^, because invasion effects are often species-, ecosystem- and context- specific^6, 7^.

Invasive plants can change soil conditions and reconstruct soil microbial community once they invade successfully, and therefore obtain soil nutrients easier than their co-existing competitors^6, 8^. Although soil microbial community may be structurally robust to environmental changes^9^, establishment of invasive plants can alter the soil microbial community composition^10–12^ and abundance of several microbial functional groups^13–15^. This could be due to invasion-induced changes in plant or/and soil properties. Invasive plants could have higher growth rate and productivity and therefore high C inputs into soils through litterfalls and root exudates to feed soil microorganisms^16–18^, with generally higher quality of substrates containing relatively high N content and low lignin content^14^. Different species exhibit eutrophic or oligotrophic characteristics and preferably utilize different kinds of substrate^19^, therefore resulting in altered soil microbial community compositions by plant invasion. Moreover, invasive plants could change a range of soil properties, e.g., soil pH value and organic C content^12, 16^, which are critical to modify soil microbial communities^12, 20^. In association with the changed microbial community composition^21^, soil microbial community functions (including soil enzyme activities involved in nutrient cycling) may also be modified^10, 12^. Consequently, these variations in soil microbial properties could induce changes in microbial community functioning such as C/N cycling and litter decomposition^11, 13^ and nutrient supplies under invasion^22^. Probably, such plant-soil interactions facilitate plant invasions^23, 24^ and thus invasion effects on soil microbial communities have received much attention.

Previous studies showed that invasive plants maintained higher growth activity^25^ and could therefore provide more substrate inputs into the soil for microbial activities. There exist reports that invasive species (e.g., *Ambrosia artemisiifolia* L. and *Eupatorium adenophorum*) could exhibit positive effects on soil microbial functions including C utilization capacity and extracellular enzyme activities, relative to their competitors^10, 22^. In a preliminary experiment, we found that the invasive *Bidens pilosa* could maintain a higher soil microbial biomass than the non-invasive *B. bipinnata* (455.4 ± 50.9 mg kg^−1^ vs. 322.5 ± 35.3 mg kg^−1^, respectively; see Fig. S1). These observations suggest that growths of invasive and non-invasive species could exert different effects on soil microbial community. Therefore, we expected that the invasive *B. pilosa* would maintain higher soil microbial activities than the non-invasive *B. bipinnata*. This could facilitate a successful invasion of alien species in new habitats with high nutrient cycling and supplies^24^.

Light availability is critical to a successful plant invasion^26, 27^. Relative to their competitors, invasive plants often have higher plasticity to obtain resources that are necessary for their growth^28^. They can maximize the aboveground biomass to compete for light in a nutrient-rich but light-limited environment, or invest more resources to produce the belowground biomass for nutrient absorptions in case that nutrients are limited^29^. As a result, invasive plants could outcompete their counterparts by adjusting the above- and below-ground C allocation very rapidly^28, 30^ or maintaining higher growth rate due to higher photosynthetic capacity at a low respiratory cost with C allocation unaltered^31^. Moreover, invasive species could reduce acceptors of light and electron transport and photochemical quantum yield through allelopathic interference and thus affect growths of those indigenous plants^32^. These changes may result in different inputs of organic materials to the soil by changing litterfall productivity and root exudates, and consequently maintain diverse soil microbial communities under different light intensities. Previous literature reported that invasive and non-invasive species could shade different soil microbial community, but whether these differences will be maintained under changed light conditions is still unclear. In this study, different levels of light intensity were set to observe responses of the soil microbial community functions under the invasive and non-invasive species. As light intensity decreased, plants may invest more resources to produce the aboveground components for the acquisition of light^29^ and consequently affect the belowground system to a less extent. Therefore, we expected that differences of soil microbial community functions under the invasive and non-invasive species would turn smaller.

Two Bidens species (*B. pilosa* and *B. bipinnata*) were chosen to use in the present study for comparisons. Although the both species originate from tropical America and has been widely introduced across tropics^33^ (also referring to Global Biodiversity Information Facility [http://www.gbif.org/species/3105856]), the former has been reported to extend very rapidly in pan-tropical zone and exert adverse effects on invaded ecosystems^32, 34^ and therefore listed as a noxious invasive weed (referring to Global Invasive Species Database [http://www.iucngisd.org/gisd/speciesname/Bidens+pilosa] and Invasive Species Compendium [http://www.cabi.org/isc/datasheet/9148]), whereas the latter has been naturalized in introduced regions^35, 36^ and regarded as an important plant resource, e.g., as a medical material in China for a long time^37^. The two species can grow well in full sun when free of disturbances. Previous studies showed that light availability greatly affected some plant traits (such as weed germination and seedling growth rate^31, 38^) and the environmental consequences differently between invasive and non-invasive species^32^. A recent study showed that the photosynthetic rate and apparent quantum yield of *B. pilosa* and *B. bipinnata* were comparable, with several traits and function differently^35^. Whether the invasive *B. pilosa* and non-invasive *B. bipinnata* affect soil microbial community functions differently in introduced habitats and whether the pattern can be maintained, however, remained rarely studied.

To compare the consequences by growing invasive and non-invasive species, this study was conducted in a subtropical garden soil of southern China, with the invasive *B. pilosa* and non-invasive *B. bipinnata* planted. The primary objectives were: 1) to compare the soil microbial community functions and nutrient availability under the two species and 2) to test whether potentially different effects of the two species would turn smaller.

## Results

### Effects on soil microbial C utilization pattern

**Figure 1:**
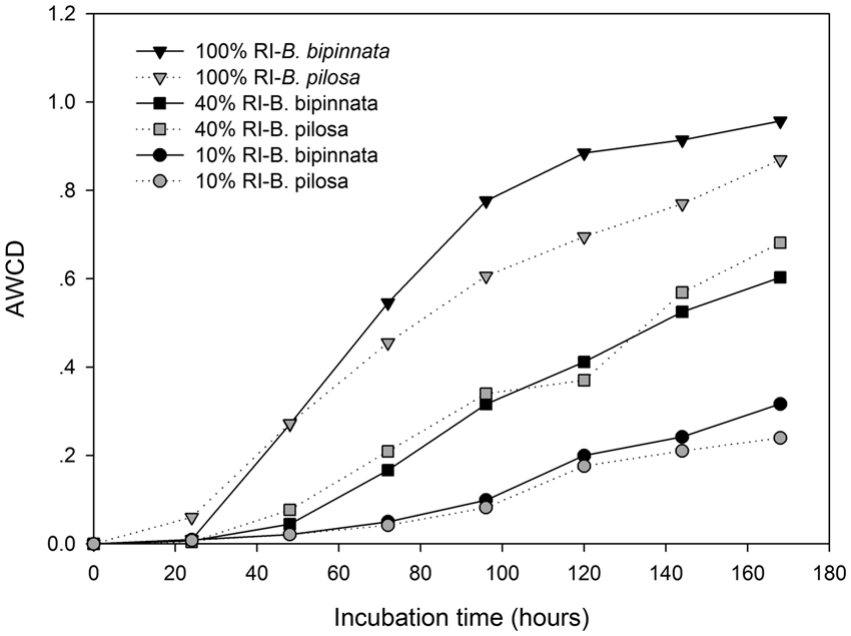
Average well color development (AWCD) of soil microbial community under the invasive *Bidens pilosa* and the non-invasive *B. bipinnata* at different levels of light intensity. Different signals indicate the three RI treatments which are at natural condition and shaded at 40% and 10% of full light intensity, respectively. At each of the light intensity treatments, black signals are the observations under *B. bipinnata* while gray signals are those under *B. pilosa*.

**Figure 2:**
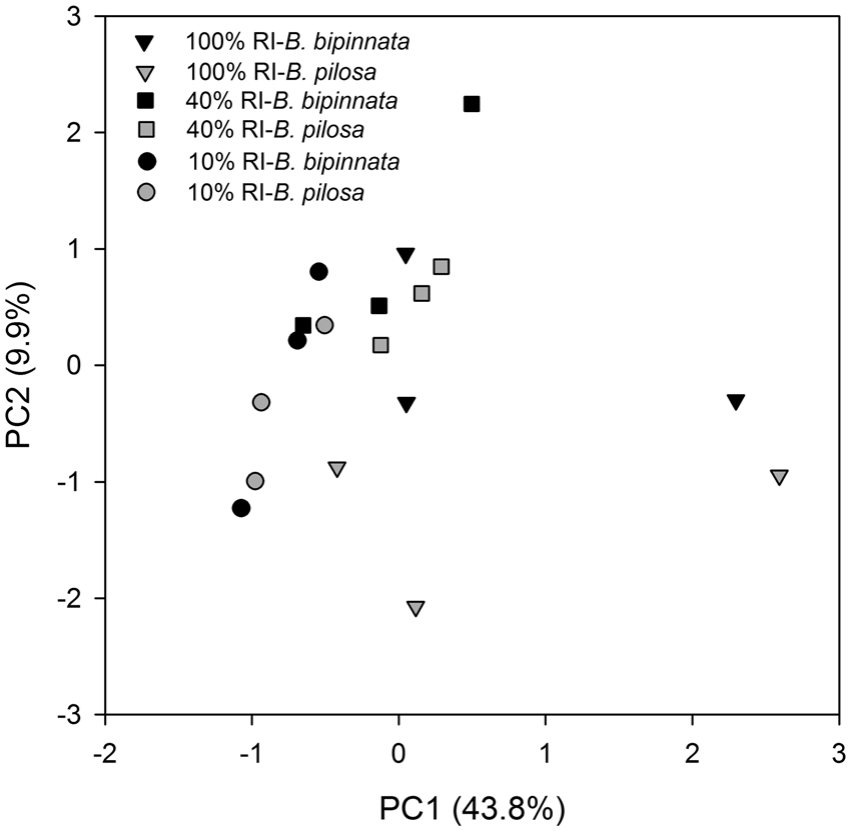
Scores of the first two principal components identified by principal component analysis on the carbon utilization pattern of soil microbial community. Different signals indicate the three RI treatments which are at natural condition and shaded at 40% and 10% of full light intensity, respectively. At each of the light intensity treatments, black signals are the observations under *B. bipinnata* while gray signals are those under *B. pilosa*.

Under full light intensity (100% RI) treatment, the invasive *B. pilosa* shaped the soil microbial communities with relatively lower microbial C utilization activity, as indicated by the lower AWCD under *B. pilosa* than that under the non-invasive *B. bipinnata* (Fig. 1). Under medium light intensity (40% RI) treatment, however, such a pattern was not maintained; an opposite trend appeared with slightly higher microbial C utilization activity under the invasive plant than its non-invasive congener. Growing *B. bipinnata* tended to contain soil microbial communities with relatively higher C utilization activity when light intensity was extremely low at 10% of full light intensity (Fig. 1). Nevertheless, plots did not significantly separate from each other between the two species or among the light intensity treatments (*p* > 0.05, Fig. 2).

**Figure 3:**
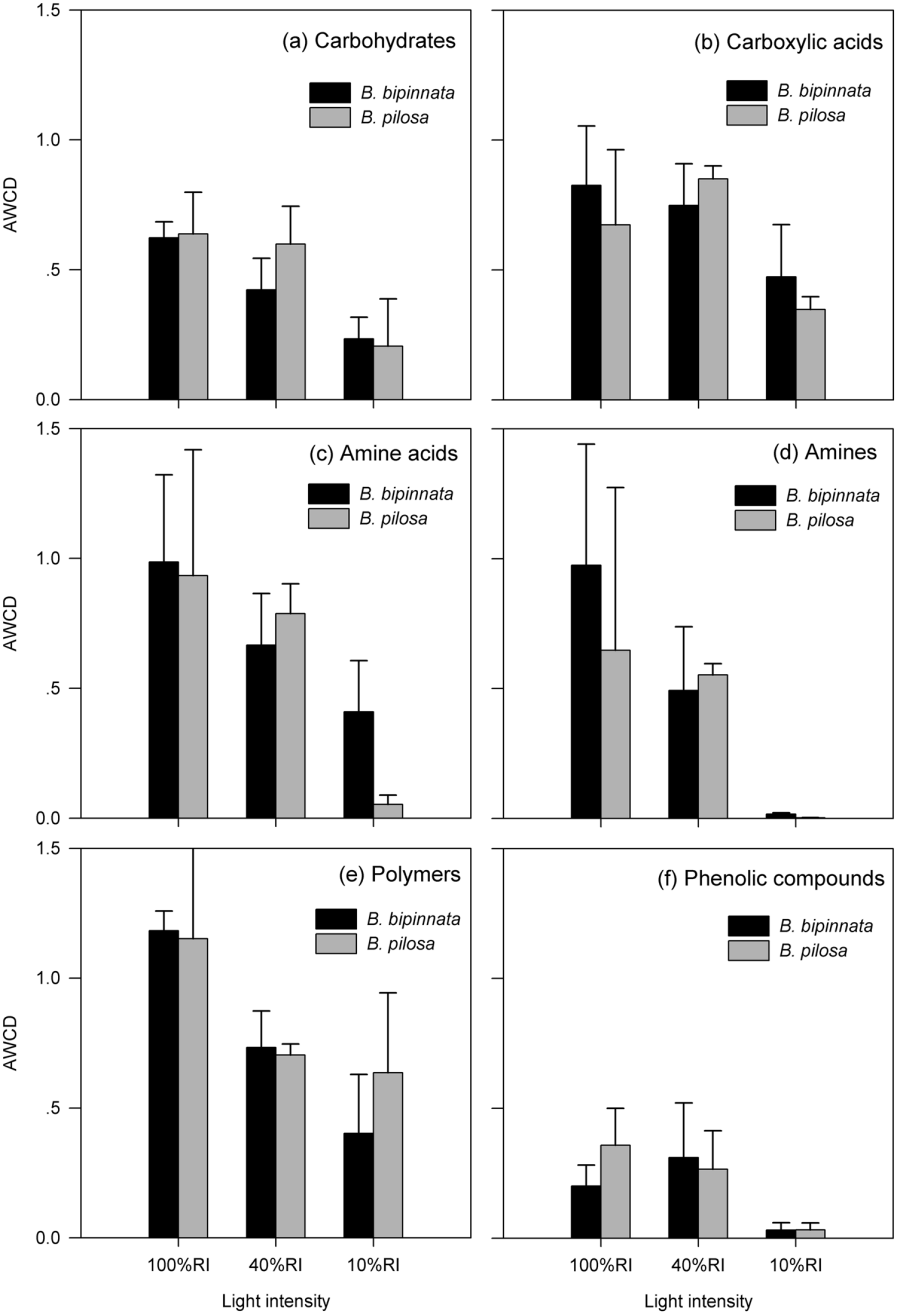
Average well color development (AWCD) of the soil microbial community to utilize different groups of carbon source in the Biolog Eco-plate at the three RI treatments. Bars stand for means and error bars are standard errors (n = 3).

The AWCD of six groups of C substrate contained in the Biolog Eco-plate, i.e., carbohydrates, carboxylic acids, amine acids, amines, polymers and phenolic compounds, was further compared between the two plant species and among the three light intensity conditions. For all the six groups of C substrates, the microbial C utilization pattern was not significantly different between plants under each of the light intensity treatments (*p* > 0.05 for all, Fig. 3). As light intensity decreased, however, the soil microbial community significantly or marginally significantly decreased C substrate utilization, as indicated by the lower AWCD under low light intensity (*p* = 0.015 for carbohydrates, *p* = 0.077 for carboxylic acids, *p* = 0.032 for amine acids, *p* = 0.073 for amines, *p* = 0.055 for polymers, and *p* = 0.078 for phenolic compounds; Fig. 3).

### Effects on soil enzyme activities

**Figure 4:**
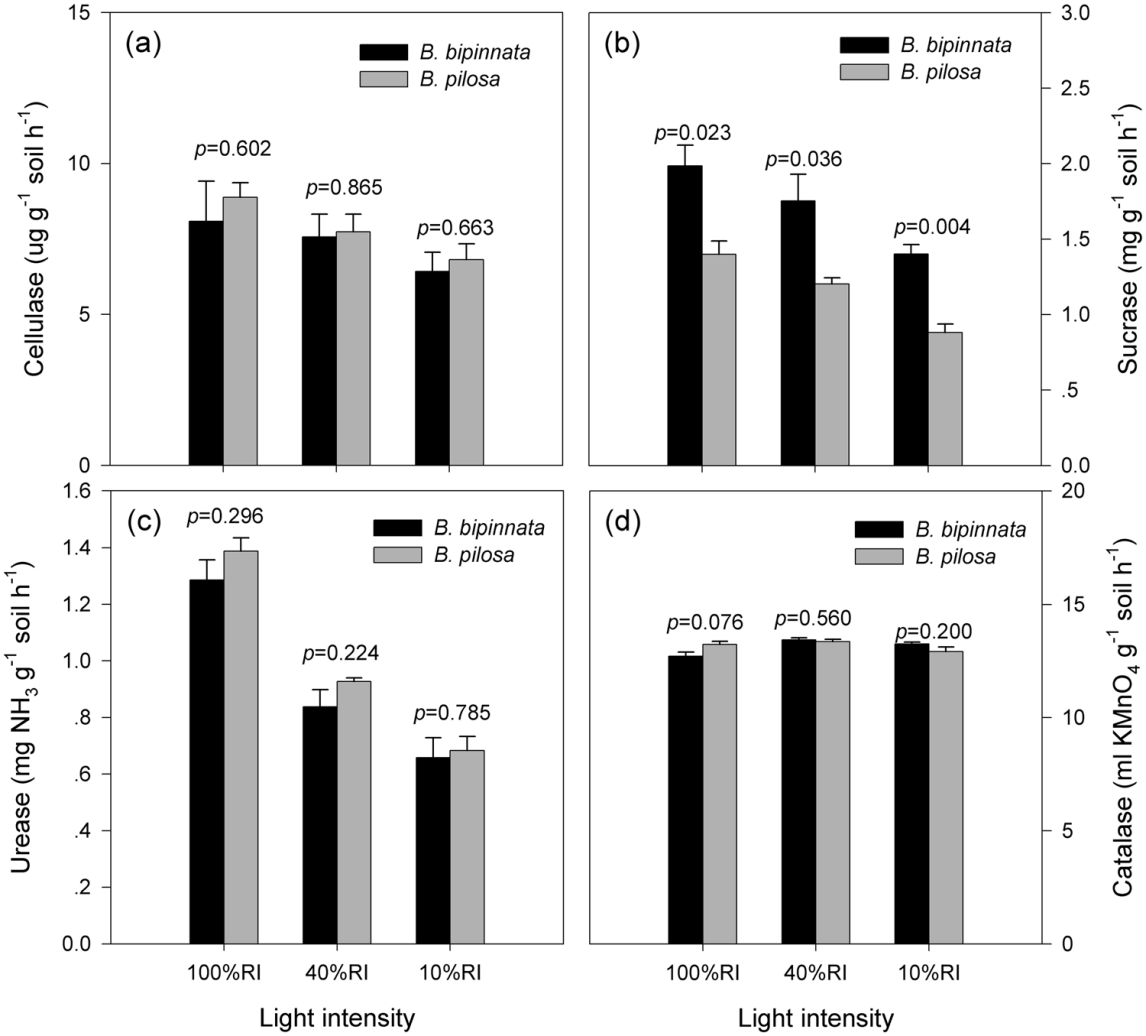
Soil enzyme activities under the two species at 100%, 40% and 10% RI treatments. Bars stand for means and error bars are standard errors (n = 3). Significance levels are indicated by the *p* values above bars at each of the RI treatments.

Soil invertase activity was significantly lower under the invasive *B. pilosa* than under *B. bipinnata* (*p* < 0.05 for all the three light intensity treatments). The other three soil enzyme activities, i.e., urease, catalase and cellulase activities, were not significantly different between the two species (*p* > 0.05 for all, Fig. 4). For the both species, soil enzyme activities were depressed when light intensity decreased (Fig. 4). However, the changes of light intensity did not significantly alter the pattern of three of the four soil enzyme activities (except catalase with *p* = 0.025) between species, as indicated by the non-significant interactions between plant species and light intensity treatment (*p* > 0.05, Table 1).

**Table 1:** Summary of two-way analysis of variances on different groups of C substrates, soil enzyme activities and soil available nutrients.

	Species			Light intensity			Interaction		
	df	F	p	df	F	p	df	F	p
Carbohydrates	1	0.001	0.980	2	4.652	0.032	2	0.153	0.860
Carboxylic acids	1	0.149	0.706	2	2.597	0.116	2	0.282	0.759
Amine acids	1	0.188	0.672	2	3.776	0.053	2	0.399	0.680
Amines	1	0.118	0.737	2	2.943	0.091	2	0.190	0.829
Polymers	1	0.059	0.812	2	2.574	0.117	2	0.135	0.875
Phenolic compounds	1	0.291	0.599	2	3.022	0.086	2	1.301	0.308
Cellulase	1	0.516	0.486	2	2.928	0.092	2	0.085	0.919
Invertase	1	41.328	<0.001	2	13.964	0.001	2	0.052	0.949
Urease	1	2.542	0.137	2	74.924	<0.001	2	0.279	0.761
Catalase	1	0.104	0.752	2	4.898	0.028	2	5.084	0.025
AHN	1	13.332	0.003	2	22.233	<0.001	2	1.123	0.357
Available P	1	5.166	0.042	2	2.621	0.114	2	0.839	0.456
Available K	1	1.794	0.205	2	245.91	<0.001	2	2.550	0.119

In the table, statistical *F* and significance level *p* with degree of freedom *df* are presented. AHN stands for alkali-hydrolysable nitrogen and P and K stand for phosphorus and potassium, respectively.

### Effects on soil available nutrients

**Figure 5:**
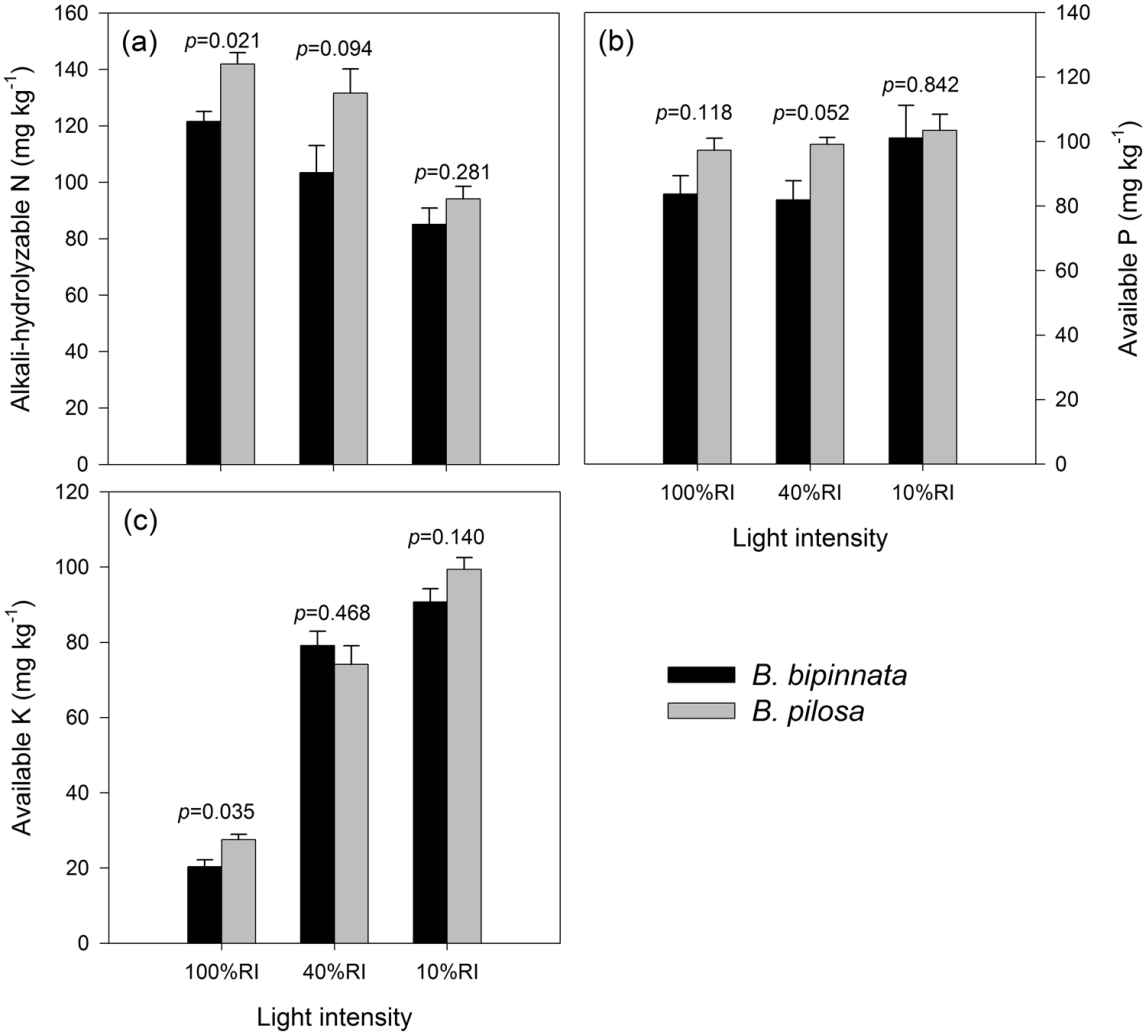
Soil available nitrogen (N; **a**), phosphorus (P; **b**) and potassium (K; **c**) contents under the two species at 100%, 40% and 10% RI treatments. Bars stand for means and error bars are standard errors (n = 3). Significance levels are indicated by the *p* values above bars at each of the RI treatments.

With full light (100% RI) treatment, soil available N and K contents were significantly higher under *B. pilosa* than under *B. bipinnata* (*p* = 0.021 and 0.035, respectively; Fig. 5a,c), but soil available P was not significantly different between the two species (*p* = 0.118; Fig. 5b). Under 40% and 10% RI treatments, the differences of soil available N, P and K contents were not statistically significant between the two species (*p* > 0.05 for all; Fig. 5), although two-way ANOVAs showed that the interactions between plant species and light intensity were not statistically significant (*p* > 0.05 for all, Table 1). Regardless of plant species, a decrease in light intensity reduced soil available N content but increase soil available P and K content (Fig. 5).

## Discussion

In the present study, the soil microbial C utilization pattern and three of the four extracellular enzyme activities were not significantly different by growing the two species (Figs 2–4). Although both originate from tropical America and have distributed in the pan-tropics, *B. pilosa* has been classified as a noxious invasive species that can extend rapidly and exhibit detrimental effects in introduced ecosystems^32, 34^ whereas *B. bipinnata* instead has been regarded as an important plant resource^37^. Our result suggests that alterations of microbial C and N utilizations could not be the reasons that result in severe invasion of *B. pilosa* in southern China^34^, compared with non-invasive congeners. Plant-soil interactions have been proposed as one of the potential mechanisms to explain a successful invasion of alien plant species in new habitats^4, 6, 23^. Although the soil microbial community has functional and structural plasticity which could make them resistant to environmental changes^9^, an increasing quantity of evidences demonstrate that plant invasions can modify soil microbial community composition^10, 11, 13^ and consequently change ecosystem processes such as litter decomposition and N cycling^3, 11, 13^. From an aspect of C and N cycling, invasive and non-invasive species did not shape significantly different soil microbial communities. However, our observation did not deny the possibility that plant-soil interactions may benefit to *B. pilosa* invasion in other ways, such as altering soil microbial community composition, increasing soil microbial biomass (also referring to Fig. S1) and stimulating soil enzyme activities^12, 39^. These may be different strategies to accelerate nutrient cycling as needed and thus to reinforce invasiveness of alien species in introduced ecosystems^8, 24^.

Although most of microbial community functions were comparable, available soil N and K contents were significantly higher under *B. pilosa* than under *B. bipinnata* in the 100% RI control (Fig. 5). This is likely associated with changes in multiple aspects of change in soil and plant characteristics, such as soil microbial biomass (Fig. S1) and nutrient contents in litter and fine root^40, 41^. Growing *B. pilosa* could substantially increase soil enzyme activities^39^ and similar positive effects were also reported for other invasive species^42^. Soil microbial biomass and enzyme activities may contribute to higher soil nutrient supplies under *B. pilosa*. More specifically, the alkali-hydrolyzable N content was significantly higher under the invasive *B. pilosa* than under the non-invasive *B. bipinnata* in the 100% RI control, which is accompanied with relatively higher soil microbial biomass and urea activity (Figs S1 and 4c).

Invasive species often have greater productivity than those non-invasive counterparts with higher capacity to tolerate environmental stresses^25^ and consequently could produce higher nutrient inputs into the soil. These processes could result in increases in soil nutrient contents such as soil N and P^3, 40, 41^, which is supported by our observations. When living in a resource-limited habitat with native and non-invasive species, invasive species may outcompete their competitors through rapid nutrient absorptions due to their fast-growth property^41^. Therefore, limited resource availability stresses and excludes those non-invasive species that have relatively lower reproduction and growth rate. This is a potential explanation for the invasion success of *B. pilosa* in southern China. At the preliminary stage, establishment of *B. pilosa* could change soil conditions (e.g., to activate more soil nutrients for its growth) to facilitate its invasion^8^. Invasive species could outcompete their competitors and invade successfully because they often have higher phenotypic plasticity and growth potential^28^. Nutrients fixed in plant tissues are returned to the soil through litterfall and root exudates, thus leading to increased contents of soil nutrients as observed in previous^22, 41^ and the present studies. This highlights the importance to consider potentially different effects of plant invasions with various severity on ecosystem processes and functions^16, 18^.

We expected that differences in those investigated variables would be smaller between the two species, because both species could prefer to invest more resources for light competition^29^ and therefore exhibit less effects on the belowground part. In the present study, the total and specific soil microbial activity and three of the four soil enzyme activities obviously decreased as light intensity declined (Figs 1, 3 and 4), partially supporting our expectation. This is likely attributable to the decreased soil microbial biomass under low light condition (Fig. S1). Soil under low light intensity could receive lower substrate inputs due to declined plant growth^31^ and therefore cannot maintain a comparable soil microbial community with that in full sun, or at least the active soil microbial community^26^. Previous literature reported that size and functions of the soil microbial community were greatly affected by substrate supply^43^. However, our observation that light intensity exhibits little interactions with species (Table 1) suggests that low light intensity did not alter species-induced differences in the soil microbial community functions.

Soil had significantly higher available soil N content but lower available soil K content under high RI condition, regardless of the species (Fig. 5). The soil N pattern is consistent with that of soil urease activity (Fig. 4c), implying light-induced changes in soil enzyme activities could alter soil nutrient condition as needed. Nitrogen is a critical to plant growth, because it can be utilized to produce chlorophyll^44^ that fixes atmospheric C via photosynthesis. Therefore, plants could up-regulate N need to generate more chlorophyll for C assimilation as light intensity increases^45^. Greater N need stimulates plant to affect the associated soil system to produce more soil urease that converts organic N as inorganic N^46^. Unlike N that can be fixed from the atmosphere and then returned into the soil to increase soil N content, P and K cannot be easily produced and activated by plants and soil microorganisms^47, 48^. High light intensity stimulate plant growth^31^ and thus increase the P and K needs from soils. As a result, available soil P and K contents will decline under high light intensity when the P and K elements cannot be activated as rapid as they are absorbed. Moreover, the soil beneath the invasive *B. pilosa* had significantly higher available N and K contents than that beneath its congener in full sun (Fig. 5a,c). This pattern turned non-significant when light intensity decreased, suggesting that light availability substantially impacts the species effects on available soil nutrient condition.

In summary, the soil microbial community beneath the invasive *B. pilosa* was not significantly different from that beneath the non-invasive congener *B. bipinnata* in this study, as indicated by the non-significant soil microbial C utilization and enzyme activities. However, soil contained higher available N and K contents under *B. pilosa* than under *B. bipinnata* under full sunlight but the differences turned non-significant when light intensity decreased. This result indicates that nutrient mobilizations could have contributed to the strong invasiveness of *B. pilosa*, which depends greatly on light availability in invaded ecosystems. As an important resource for plant growth, light availability substantially also changed the soil microbial community functions and available soil N and K contents. Our results suggest that nutrient mobilizations could contribute to the strong invasiveness of *B. pilosa* relative to its non-invasive congeners. Nevertheless, plant-induced alterations of microbial C utilization pattern may not be the reasons for *B. pilosa* invasion.

## Methods

### Site description and experiment preparations

This study was conducted at the experimental and teaching farm of South China Agricultural University in Guangzhou. This region has the typical subtropical monsoon climate, with annual air temperature being 21.8 °C and annual precipitation being 1694 mm^49^. Most of the precipitation occurs from April to September (the wet season). This results in an obvious dry-wet season cycling each year in the study site.

On December 2011, seeds of *B. pilosa* and *B. bipinnata* were collected from wild populations at South China Agricultural University (N 23°16′, E 113°37′) and South China Botanical Garden (N 23°18′, E 113°36′) in Guangzhou, respectively. They were dried in the sun and then stored in the sealed plastic bags at 4 °C until used for incubation (around 4 months). On March 31 2012, seeds were sowed to raise seedlings using breeding beds in a greenhouse which located at College of Agriculture, South China Agricultural University. During the period, seedlings were thinned to leave enough space for the growth of each seedling after 20 days of culturing seedlings. Ten days after thinning, uniform individuals of *B. pilosa* and *B. bipinnata* which were approximately 10 cm high were transplanted in pots for the following study.

### Experimental design

Sun-shelters with a size of 4 × 4 m^2^ were established for light intensity treatments, by means of covering black shading net with different light transmissions in the experimental and teaching farm of South China Agricultural University. Finally, three levels of relative light intensity, i.e., two sheltering treatments with 40% and 10% relative intensity (RIs) of full sunlight and the full sunlight control (100% RI), were established to explore potential roles of light availability on plant-induced effects on the soil microbial community functions and nutrient contents.

Under each light intensity treatment, three pots (40 cm diameter × 33 cm height) were used to grow seedlings of the invasive *B. pilosa* and another three used to grow seedlings of its congener *B. bipinnata*. Each pot contained 7.5 kg soil, with two individuals of each species grown. The used soil was collected from the experimental and teaching farm of South China Agricultural University in which both of the two species grew and then composited completely for the following plant cultivation. The soil organic matter was 2.1% and soil available nitrogen (N), phosphorus (P) and potassium (K) were 120.8, 96.0 and 99.7 mg kg^−1^, respectively. Plantation duration was 64 days and through the period, all the pots were watered per day to maintain soil water content. At the end of conditioning phase, soil samples were collected to analyze for soil microbial properties and available N, P and K contents.

### Soil analyses

The assayed soil microbial community properties include microbial carbon (C) utilization pattern and soil enzyme activities. Soil microbial C utilization pattern was determined using BIOLOG EcoPlate^TM^ (Biolog Inc., CA, USA) which contains 31 types of C substrate commonly used by soil microorganisms and one substrate-free control^50^. For each sample, 10 g fresh soil was placed into a sterilized glass flask to mix with 100 ml of 0.85% sterilized NaCl solution on a reciprocal shaker for 0.5 h and then let stand for 1 h. The supernatant was diluted 1000 times and then 150 μl of the diluted soil suspension were added into each well of the Biolog Eco micro-plate. The micro-plates were incubated for 7 days at 25 ^o^C and optical density was read at 590 nm using a Biolog Gen III Microstation (Biolog Inc., CA, USA) per day to record the color development of soil samples^51^. Finally, average well color development (AWCD) was calculated to indicate microbial C utilization for each sample. This method can clearly exhibit changes in soil microbial community function to utilize C substrates^50^ and the results to some extent indicate the similar changes of soil microbial community, e.g, profiled by phospholipid fatty acid analysis^52^.

The activities of four soil enzymes including cellulase, invertase, urease and catalase were also analyzed to indicate soil microbial community functions^10, 53^, following the methods proposed by Guan^53^ and Yao and Huang^54^. Briefly, soil cellulase and invertase activities were determined by the dinitrosalicylic acid (DNS) reduction and colorimetric method, with 1% carboxymethylcellulose and 8% sucrose solutions as substrates, respectively^53, 54^. Soils mixed with the according substrate and phosphate buffer solution were incubated at 37 ^o^C for 72 h to analyze soil cellulose activity and for 24 h to analyze soil invertase activity. The DNS solution was then added to develop color for 15 min. Finally, color density was read at 540 and 508 nm to calculate soil cellulase and invertase activity, respectively. Urease activity was analyzed using the phenol-hypochlorite reaction and colorimetric method, with 10% urea solution as a substrate^54^. After incubated at 37 ^o^C for 24 h, phenol and hypochlorite solutions were added to develop color and then color density was read at 578 nm. Catalase activity was tested by the KMnO_4_ oxidation and titration method, with 0.3% H_2_O_2_ solution as a substrate and 0.02 mol L^−1^ KMnO_4_ as an oxidizing reagent^53^.

Soil available N, P and K contents were determined as described by Bao^55^. Soil available N was assayed using the alkaline hydrolysis-diffusion method, i.e., the available N was reduced to NH_3_ at 40 ^o^C for 24 h after adding FeSO_4_ powder and a NaOH solution and then the NH_3_ was absorbed using H_3_BO_3_ and titrated using H_2_SO_4_ to determine soil available N content. Available P content was determined using a spectrophotometer at the wavelength of 700 nm, with 0.05 mol L^−1^ HCl-0.025 mol L^−1^ (1/2 H_2_SO_4_) as the extractant, ascorbic acid as the reduction agent and a H_2_SO_4_-(NH_4_)_6_Mo_7_O_24_ solution as the color development agent. Soil available K was extracted by 1 mol L^−1^ NH_4_OAc solution and K concentration in the extracts was determined by the flame spectrometry method. Soil available N, P and K contents were presented as mg kg^−1^ soil in this study.

### Statistical analyses

Principal component analysis (PCA) was conducted to reveal overall treatment effects on the microbial C utilization pattern originated from BIOLOG analysis. The PCA results could visually present the treatment effects. Moreover, the 31 types of C substrate in BIOLOG EcoPlate were pooled into six groups according to their properties^50^. For each group of the C substrates, AWCDs were compared between species and among light intensity treatments using independent-samples *t* test and one-way analysis of variance (ANOVA), respectively. The independent-samples *t* test and one-way ANOVA were also used to detect significant differences in soil enzyme activities and available N, P and K contents among treatments. Two-way ANOVA was employed to test the main and interactive effects of plant species and light intensity on microbial C utilization pattern, soil enzyme activities or available nutrient contents. For all the statistical analyses, significance level was set at *p* < 0.05. All these analyses were conducted in IBM SPSS Statistics 22 (IBM Corp., NY, USA) and graphs were made in SigmaPlot 10.0 (Systat Software Inc., CA, USA).

### Data availability statement

The datasets generated during the current study are available from the corresponding author on reasonable request.

## Electronic supplementary material

Supplementary figure 1
