# Supplementary material for: Soil microbial carbon utilization, enzyme activities and nutrient availability responses to Bidens pilosa and a non-invasive congener under different irradiances
Source: Sci Rep. 2017 Sep 12;7:11309. doi: 10.1038/s41598-017-11707-x (PMC5596010; doi:10.1038/s41598-017-11707-x)

SUBJECT AREAS: Ecology, Environmental Sciences

Correspondence and requests for materials should be addressed to J.Z. (jeanzh@scau.edu.cn)

Soil microbial carbon utilization, enzyme activities and nutrient availability responses to *Bidens pilosa* and a non-invasive congener under different irradiances

Hui Wei [1,2,3], Wenbin Yan [1], Guoming Quan [3,4], Jiaen Zhang [1,2,3,*], Kaiming Liang [3,5]

[1] Department of Ecology, College of Natural Resources and Environment, South China Agricultural University, Guangzhou 510642, China

[2] Key Laboratory of Agro-Environment in the Tropics, Ministry of Agriculture, Guangzhou 510642, China

[3] Guangdong Engineering Research Center for Modern Eco-agriculture and Circular Agriculture, Guangzhou 510642, China

[4] Department of Urban Construction Engineering, Guangzhou City Polytechnic, Guangzhou 510405, China

[5] The Rice Research Institute of Guangdong Academy of Agricultural Sciences, Guangzhou 510640, China

[*] Corresponding author: Prof. Jiaen Zhang

College of Natural Resources and Environment, South China Agricultural University

483 Wushan Road, Tianhe District

Guangzhou 510642

China

Tel.: + 86 20 8528 0211

Fax: + 86 20 8528 1885

Email: jeanzh@scau.edu.cn

Fig. S1 | Soil microbial biomass carbon (C) under the two species at 100%, 40% and 10% RI treatments. Bars stand for means and error bars are standard errors (n=4).

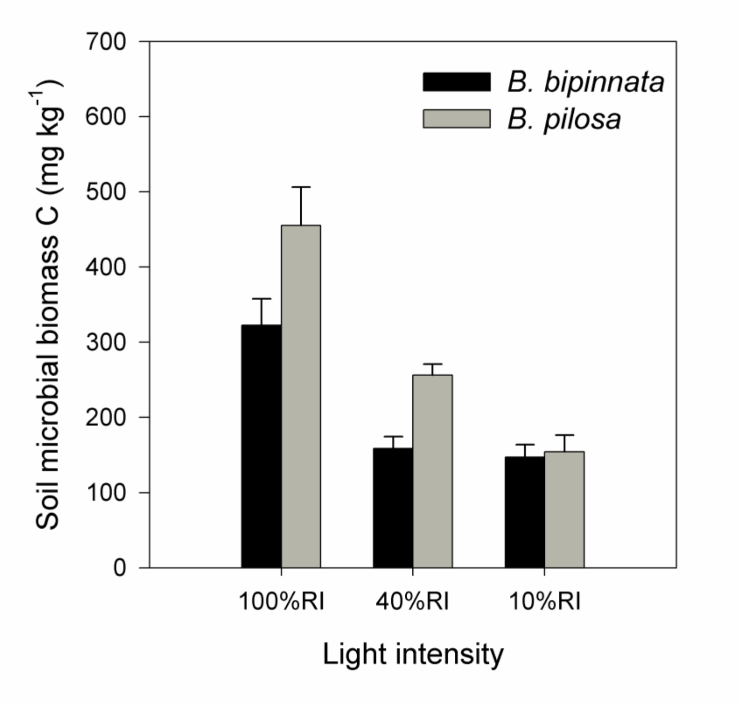

Supplement: Supplementary file 1 — Supplementary figure 1 [file 41598_2017_11707_MOESM1_ESM.pdf]
